# Effects of rest intervals on lower extremity kinematics and coupling during the Star Excursion balance test

**DOI:** 10.1186/1757-1146-7-S1-A49

**Published:** 2014-04-08

**Authors:** Yongung Kwon, Dorsey S Williams

**Affiliations:** 1 Department of Health and Human Performance, Virginia Commonwealth University, Richmond, VA, 23284, USA; 2 Department of Physical Therapy, Virginia Commonwealth University, Richmond, VA, 23298, USA

## Background

Kinematic differences exist in ankle joint motion between individuals with and without chronic ankle instability (CAI) and have been recognized during walking, running and jumping[1]. The Star Excursion Balance Test (SEBT) is a common test used to evaluate dynamic postural control by measuring reach distance[2]. However, little is known regarding lower extremity joint motion and coupling during this task and regarding the between trial rest interval and its potential relationship to fatigue and kinematics. Therefore, the purpose of this study is to investigate lower extremity kinematics and coupling relationships during the SEBT at different rest intervals.

## Methods

Seven male and 8 female subjects without a history of ankle sprains participated. The order of rest intervals (10, 20, 40 seconds) and reach direction (AM;anteriormedial, M:medial, PM:posteriormedial) were counterbalanced. A total of three visits were required. Subjects performed 7 consecutive trials of the SEBT in each of the 3 directions. The final 3 trials were used for analysis. Initial and peak ankle joint angles of eversion(EV), dorsiflexion(DF), and tibial internal rotation(TIR) were measured using three-dimensional motion analysis. Excursions and coupling angles were calculated for each individual and ensemble averages were created. Two-factor analyses of variance were used to compare excursions of DF, EV and TIR and coupling ratios of TIR/DF and TIR/EV across the 3 directions in the 3 rest interval groups.

## Results

There were no significant differences for any variables across rest intervals. Differences existed across directions only (Table 1). There were no interactions on any variables.

**Table 1:** Mean(sd)° across directions

	AM	M	PM	p value (ANOVA)
Excursion				

EV	4.9(2.9)	4.7(2.8)	4.9(3.0)	0.90
DF	13.2(5.1) *^±^	11.8(5.5) ^¥^	9.3(5.4)	<0.01
TIR	-6.7(2.6)	-8.2(4.7) ^*^	-8.5(4.0) ^±^	0.05

Ratio				

TIR/DF	-0.6(0.4)	-0.9(0.8) *	-1.6(2.3) ^±¥^	<0.01
TIR/EV	-2.4(6.2)	-2.4(3.1)	-2.8(3.4)	0.93

p<0.05: *AM vs. M, ^±^AM vs. PM, ^¥^M vs. PM

## Conclusions

Different intervals of rest ranging from 10 to 40s did not influence ankle angular excursions or coupling ratios during the SEBT in a healthy population. There is a progressively decreased demand for ankle DF when moving from AM to PM. Further, TIR of ankle in AM occurs less than in both M and PM. Based on these results, DF, TIR, and the coupling of these motions may play an important role in dynamic postural control as measured by the 3 directions of the SEBT. Future studies will focus on the comparison of healthy subjects and those with CAI.

## References

[B1] Kipp K Palmieri-Smith RM Differences in kinematic control of ankle joint motions in people with chronic ankle instability Clinical Biomechanics 2013 23 562 567 23601918 10.1016/j.clinbiomech.2013.03.008

[B2] Olmsted LC Carcia CR Hertel J Shultz SJ Efficacy of the Star Excursion Balance Tests in detecting reach deficits in subjects with chronic ankle instability Journal of Athletic Training 2002 37 501 506 12937574 PMC164384

